# NAVIS-An UGV Indoor Positioning System Using Laser Scan Matching for Large-Area Real-Time Applications

**DOI:** 10.3390/s140711805

**Published:** 2014-07-04

**Authors:** Jian. Tang, Yuwei. Chen, Anttoni. Jaakkola, Jinbing. Liu, Juha. Hyyppä, Hannu. Hyyppä

**Affiliations:** 1 GNSS Research Center, Wuhan University, 129 Luoyu Road, Wuhan 430079, China; E-Mail: tangjian@whu.edu.cn; 2 Department of Remote Sensing and Photogrammetry, Finnish Geodetic Institute, Kirkkonummi FI-02431, Finland; E-Mails: anttoni.jaakkola@fgi.fi (A.J.); jingbin.liu@fgi.fi (J.L.); juha.hyyppa@fgi.fi (J.H.); 3 Department of Real Estate, Planning and Geoinformatics, Aalto University, Espoo FI-11000, Finland; E-Mail: hannu.hyyppa@aalto.fi

**Keywords:** laser scan matching, indoor position, real-time, iterative closed point, unmanned ground vehicle

## Abstract

Laser scan matching with grid-based maps is a promising tool for real-time indoor positioning of mobile Unmanned Ground Vehicles (UGVs). While there are critical implementation problems, such as the ability to estimate the position by sensing the unknown indoor environment with sufficient accuracy and low enough latency for stable vehicle control, further development work is necessary. Unfortunately, most of the existing methods employ heuristics for quick positioning in which numerous accumulated errors easily lead to loss of positioning accuracy. This severely restricts its applications in large areas and over lengthy periods of time. This paper introduces an efficient real-time mobile UGV indoor positioning system for large-area applications using laser scan matching with an improved probabilistically-motivated Maximum Likelihood Estimation (IMLE) algorithm, which is based on a multi-resolution patch-divided grid likelihood map. Compared with traditional methods, the improvements embodied in IMLE include: (a) Iterative Closed Point (ICP) preprocessing, which adaptively decreases the search scope; (b) a totally brute search matching method on multi-resolution map layers, based on the likelihood value between current laser scan and the grid map within refined search scope, adopted to obtain the global optimum position at each scan matching; and (c) a patch-divided likelihood map supporting a large indoor area. A UGV platform called NAVIS was designed, manufactured, and tested based on a low-cost robot integrating a LiDAR and an odometer sensor to verify the IMLE algorithm. A series of experiments based on simulated data and field tests with NAVIS proved that the proposed IMEL algorithm is a better way to perform local scan matching that can offer a quick and stable positioning solution with high accuracy so it can be part of a large area localization/mapping, application. The NAVIS platform can reach an updating rate of 12 Hz in a feature-rich environment and 2 Hz even in a feature-poor environment, respectively. Therefore, it can be utilized in a real-time application.

## 1. Introduction

The Global Navigation Satellite System (GNSS) provides various position and navigation services in outdoor environments, including open sky areas and degraded urban areas. However, indoor navigation with the GNSS remains an unsolved challenge due to the low penetration of GNSS signals indoors. There is a rapidly growing market need for mobile indoor Location-Based Services (LBS) such as pedestrian navigation, first-aid application, and asset tracking. In recent years, much been done to establish a wide spectrum of techniques, such as WiFi, Bluetooth, UWB, LiDAR serving the needs of indoor positioning and navigation [1–7]. Such systems have become increasingly significant with their improved accuracy, reliability, availability, and affordability.

The SLAM (Simultaneous Localization and Mapping) problem is the process of building a map of an unknown environment by traversing it with range sensors (laser, sonar, *etc.*) mounted on an UGV, while simultaneously determining the location on the map. It combines positioning and mapping in a single framework, and it is considered to be an effective method for resolving indoor positioning and environment-recognizing problems [4–23]. There are two major strategies for determining the position in SLAM: absolute positioning with feature matching [4–6] and relative positioning with scan matching. Theoretically, the first method extracts features from range scans, such as lines and corners, then matches the extracted known features with a preset feature map to recognize the position. In relative positioning, on the contrary, scan matching utilizes two or more consecutive frames of scan points directly to obtain the UGV's movement with various algorithms, e.g., classical Iterative Closed Point (ICP) [24], Iterative Closed Line (ICL) [25,26], and Maximum Likelihood Estimation (MLE) [27–29]. Scan matching has been proved to be one of the most frequently relied-upon tools for determining a mobile robot's indoor position. Unfortunately, there is a critical real-time implementation problem when using such relative positioning technology: The nature of partly matching consecutive scans of ICP and ICL causes the heading estimation to drift quickly when the sample frequency is not high enough, and the accumulated errors will ruin the position accuracy with time, even though the processing time is less when compared with the MLE algorithm. While MLE is a global map matching method based on a grid map, the higher the resolution grid map is, the better the position result that can be calculated. However, large-area and high-resolution indoor positioning and mapping applications require huge amounts of memory to store the grid map, and such a time-consuming search for the best matching strategy results in a real-time fulfillment mission impossible for an onboard computer. Although some optimized solutions have been introduced using heuristics algorithms, such as hill climbing and Monte Carlo [28] to accelerate the search processing, it still produces inadequate results because all of the proposed methods strive to obtain local optimized matching rather than global optimized matching. This being the case, a new strategy is needed to balance the computational power and accuracy of real-time UGV positioning to support tangible large-area indoor applications such as mapping, data collecting, and navigation. The development of such technology could trigger a revolutionary advancement of map surveying, robotic navigation, rescue applications, *etc.* Therefore, it is an important component of indoor position and navigation.

In this paper, we introduce an efficient real-time mobile UGV indoor positioning system called NAVIS based on a new relative positioning strategy for laser scan matching using an Improved probabilistically-motivated Maximum Likelihood Estimation (IMLE) algorithm. Compared conventional probabilistically-motivated MLE, the improvements embodied in IMLE include: (a) Iterative Closed Point (ICP) preprocessing, which adaptively decreases the search scope; (b) a totally brute search matching method on multi-resolution map layers, based on the likelihood value between current laser scan and the grid map within refined search scope, adopted to obtain the global optimum position at each scan matching; and (c) a patch-divided likelihood map supporting a large indoor area.

The NAVIS system is composed of a data collecting hardware platform and data processing software. Simulations and field tests are based on the system, and the results have proven that with such improved process can enable our positioning system to produce more accurate and robust position results within low latency for long-term running of large-area applications. In this paper, we introduce a related laser scan matching method, an improved fusion position algorithm, an overview of our real-time system, and we then discuss in detail the results of our solution with simulated and real data.

**Figure 1.**
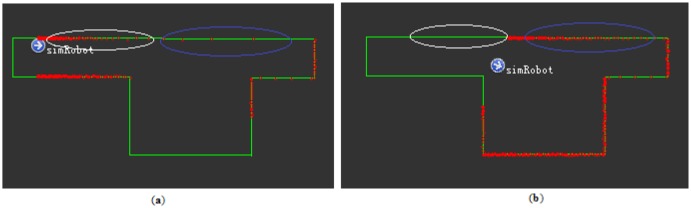
(**a**) A liDAR scan S_t−1_ at position x_0_. (**b**) A liDAR scan S_t_ at position x_1_.

As introduced in the above, laser scan matching is a relative positioning method using laser scanners. Consider a UGV moving in a flat indoor environment from position x_0_ to x_1_; at each position, it obtains a laser scan (S_0_ and S_1_) about the environment. It is then possible to find a rigid-body transformation T that projects S_t_ to align with S_t-1_. This process of matching is known as scan matching (see Figure 1). In a 2D environment, T is composed of (Δx, Δy, Δθ), representing the UGV's motion at translation and heading. The major challenge of scan matching is to minimize the runtime complexity while obtaining highly accurate position data.

As mentioned in the above, ICP and ICL have been widely adopted as fast scan matching methods utilizing least squares to find the correspondences for all points in two consecutive scans. However, as is shown in the parts circled in white in Figure 1, a number of points in the scan on the left may have no corresponding points on the scan on the right. In such a situation, the ICP algorithm will produce deficient result, especially as regards heading estimation. The ICL algorithm is similar to the ICP algorithm but it evolves from a point-point match strategy to a point-line match strategy. When comparing the parts circled in blue along the sparse scan line in Figure 1a, it became evident that the points of the scan circled in blue in Figure 1b matched better under the premise that a contour is extracted first. However, the uncertainty of the laser point cloud makes the result worse. If simply connecting the scan points in the dense part of the point cloud, point uncertainty distorts the line. Some other methods, such as polar coordinates, Normal Distribution Transform (NDT), feature-based methods, Hough Transforms (HT), and histograms [29], have been adopted to extract the line feature, but they increase the computing complexity, and thus they are not suitable for real-time applications.

The map-based MLE is a probabilistic scan matching method. A grid map M stores the likelihood value created from one or more previous scans, and the incoming scans *S_t_* are matched against the map at each map cell, represented as Tα*S_t_*, to find the best body transformation T*, at which the entire scan has the maximum likelihood value. According to Bayes's rules, the likelihood for an entire scan is computed as:

(1) $$\text{P}\left(\text{St}|\text{M}\right)=\prod_{i=1}^{n}P(x_{i}|M)$$

And the rigid-body transformation is computed as:

(2) $$T^{*}=\text{argmax}(\text{P}(\text{T}\propto S_{t}|\text{M}))$$

The likelihood value P(*x_i_*|*M*) of a single point *x_i_* is proportional to the distance d to the nearest environment's feature F, according to the Gaussian probability model of laser measure noise.

(3) $$\text{P}\left(x_{i}|M\right)\propto e^{\left(-d(x_{i},F)/\sigma \right)}$$

where σ is the scale parameter of the sensor measuring noise.

Various strategies have been proposed to determine the likelihood value and search for the best rigid-body transformation based on the above theory. Konolige and Chou [30] and Olson [31] approximated the value in terms of its distance from any surface in map M and the Graphic Processing Unit (GPU) hardware-accelerated multi-resolution map-based brute search algorithm is utilized to search for the best transformation. Bachrach *et al.* [27], extracted contours like ICL's line feature, and then computed the likelihood value based on the distance from the laser points to the nearest contour, and they also provided a fast algorithm for computing the likelihood value and it utilized a map-based coordinate ascent for brute search. Steux and El Hamzaoui [28] proposed a simple occupied mark to represent the likelihood value and a Monte Carlo heuristics research was used to search for the best transformation in their “tinySLAM” system. Prior studies have proved that existing heuristics research methods can easily fall to local maxima and fail to obtain sufficient results, even though their computation costs are lower; brute search is a global search, which is a robust but time-consuming method, and it assures that the best result is found from each candidate. Therefore, we have to narrow down the search scope of the brute search method as much as possible for robust real-time and high-accuracy positioning applications.

A new fusion search method for real-time UGV positioning is proposed in this paper. Based on Olson's MLE method, the proposed method's main contributions are: (1) the original ICP algorithm is used for rough positioning and for narrowing down the search scope before the MLE search; (2) a multi-resolution grid map for further accelerating the maximum likelihood value search of the MLE; (3) a line-feature-based three-level strategy of likelihood determination for accurate environmental feature representation; and (4) a patch-divided likelihood map for large indoor areas.

## 2. Fusion Position Algorithm

### 2.1. Algorithm Overview

**Figure 2.**
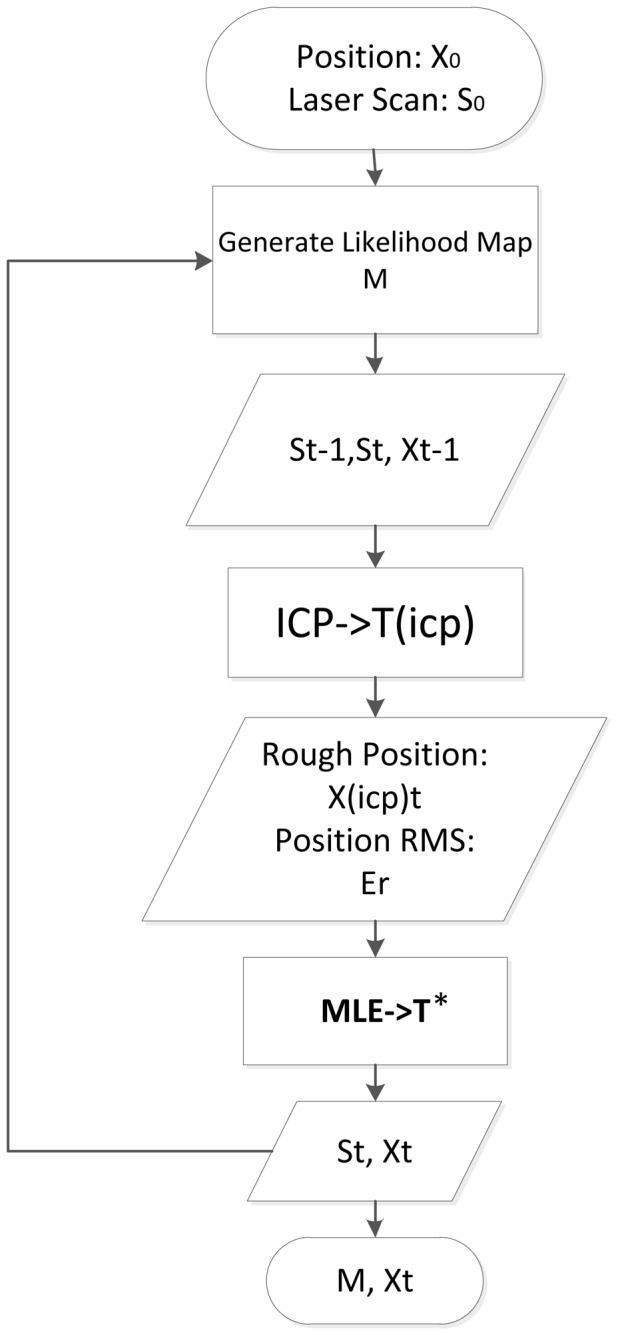
Flow chart of the fusion position algorithm.

The flow chart of our fusion positioning algorithm is shown in Figure 2. Given the initial position x_0_ and laser scan S_0_, an initial likelihood map M is generated according to the line-feature-based three-level strategy for accurate feature representation. Then an iterator of the positioning and mapping process steps in: first, the original ICP utilizes two consecutive laser scans S_t−1_ and S_t_ to calculate a rough rigid-body transformation T_icp_, then a rough position x_(icp)t_ with its corresponding RMS Er are computed out. Such parameters are used to evaluate the distance to the real position. Using this, we narrow down the search area for the MLE. Next, the MLE quickly refines the rough position making it the accurate position x_t_ in the narrowed down search area of the multi-resolution patch-divided grid map. Finally, the likelihood map M is updated with x_t_ and S_t_. The details of each part will be introduced in the following context.

### 2.2. ICP Matching Method in Estimating Rough Position

The MLE is a Nondeterministic-Polynomial-time-Complete (NPC) search problem [32]. Therefore, in order to find the best position as fast as possible, it is necessary to give an initial position and narrow down the search space. The ICP matching method is adopted with this objective in this paper, which uses least squares to find the rigid transformation of all points in two consecutive scans. The main functions of the ICP matching method are listed below:

(1) $$\text{E}=\sum_{i=1}^{k}{\left(Rp_{i}+t-q_{i}\right)}^{2}=\text{Min}$$

(2) $$\text{Er}=\text{RMS}=\sqrt{\frac{\text{E}}{k}}$$

where *R* is the rotation matrix, d*θ* is the heading angle change; *t* is the motion vector in scan *q*'s local coordinate reference, which depicts the transformation from scan *p* to scan *q* as Equations (6) and (7) present:

(3) $$\text{R}=\left\lceil \begin{array}{cc} \text{cos}(\text{d}\theta ) & -\text{sin}(\text{d}\theta )\\ \text{sin}(\text{d}\theta ) & \text{cos}(\text{d}\theta ) \end{array}\right\rceil$$

(4) $$\text{t}=[\begin{array}{cc} \text{dx} & \text{dy} \end{array}]$$

**Figure 3.**
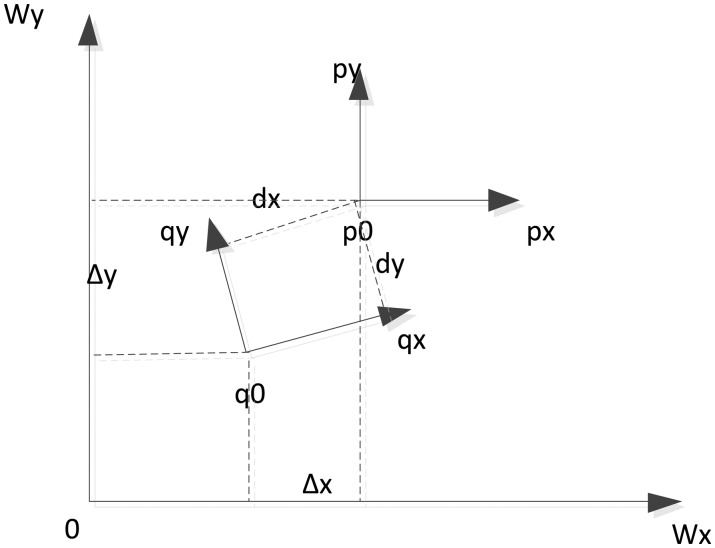
Coordinate reference relationship of ICP.

The final transformation T_(icp)_ in the global coordinate reference is:

(5) $${\text{T}}_{\left(\text{icp}\right)}=[\begin{array}{ccc} \Delta \text{x}, & \Delta \text{y}, & \Delta \theta \end{array}]=[\begin{array}{cc} {\text{R}}_{\text{g}}\text{t}, & \text{d}\theta \end{array}]$$

where R_g_ is the UGV's rotation matrix in the global coordinate reference. Figure 3 shows the relationship between the local and global map coordinate references discussed in this research.

### 2.3. Fast Generation of Likelihood Map

The likelihood map provides the background match information from the previous laser scans and it can be calculated according to Equation (3). In this paper, a simple line-feature-based and three-level strategy of likelihood value determination is proposed to generate the likelihood map M, which combines the grid-point occupation method [28] and the contour-slope method [27] to provide fast but robust scan matches.

**Figure 4.**
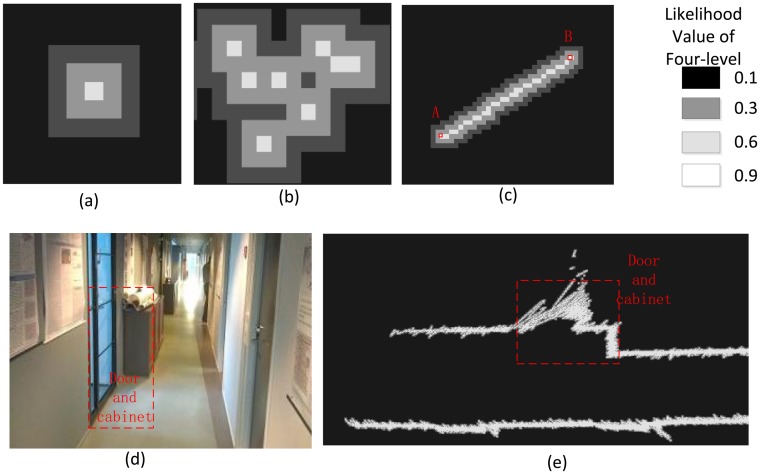
(**a**) The three-level strategy of likelihood value determination; (**b**) A greater value rule for updating the likelihood value; (**c**) The extracted line between two ordered points; (**d**) A picture of the corridor environment; and (**e**) The result likelihood map generated applying the line-feature-based three-level strategy.

The likelihood map is initialized with a grid with a given geographic boundary and resolution in the local reference. The grid cell is populated with any pre-defined likelihood values −0.1, 0.3, 0.6, and 0.9, which represent the likelihood of the environment gradually. All grid cells on the map are initialized with the likelihood value of 0.1, and each point of a new laser scan is projected onto the map grid cell. If any grid cell is occupied by a laser point, then a maximized likelihood value of 0.9 is given. We slope two grid cells around that cell and set the likelihood values as 0.6 and 0.3 outward respectively, as is shown in Figure 4a. The greater value rule is introduced to solve the overwrite conflict problem as is shown in Figure 4b. In order to solve the matching problem of spare points area in Figure 1, based on the aforementioned three-level likelihood value determination strategy, a line feature is extracted by simply connecting two ordered points within a certain distance in the laser scan, which could describe the environment more accurate. Figure 4c shows the extracted line feature from the ordered scan points A and B. Figure 4d,e are examples presenting such projection relationships in the real environment.

### 2.4. Maximum Likelihood Estimation

Given a likelihood map constructed from the previous scans using the above fast generating algorithm, a procedure is needed to search for the most likely alignment of a new laser scan to the map. A multi-level resolution grid map-based brute search method is applied in this study [27] and Algorithm 1 shows the pseudo-code of the proposed MLE search algorithm.


**Algorithm 1**: Pseudo-code of the MLE algorithm based on a multi-level resolution grid map
 **Requires:**
Search window W = [w*θ*,wx,wy];Rough position P_ICP_ = [*θ*,*x*,*y*];New scan S_t_. **Setting**: d*θ* = 0.5 degree, *dx* = *dy* = Map resolution **for** each map layer from low to high resolution  **for** each candidate position P_C_ = [*θ*+*dθ,x*+*dx,y*+*dy*] in search window W   calculate the likelihood value C of new scan S_t_ according to function (1)  **end for** **end for****return** P_Cmax_, which has maximum likelihood value in all candidates


Before this brute MLE algorithm begins the search, the ICP provides the rough position P_ICP_ and narrowed-down search window parameters-[*wx*,*wy*]. The value of w*θ* is pre-selected as 20 degrees according to the dynamics of the UGV platform. Then the likelihood of each candidate position P_C_ is calculated from the low to high resolution map layers, and P_Cmax_, which has a maximum likelihood value in all candidates, is selected as the positioning of the platform. The map layer resolution can be adaptively set based on the compromise of map creating, updating and searching. Dual-resolution map layers were adopted in this paper: the high was 1cm and the low was 5cm.

The compromise between the point number of each laser scan S_t_ and the numerical precision of the cost function as Equation (1) presented have to be determined carefully to avoid the failure of likelihood calculation caused by the overflow problem.

### 2.5. Multi-Resolution Patch-Divided Likelihood Map

The likelihood map is a typical multi-layer grid map produced using the Geographic Information System (GIS) [33]. The data size and the operation of the map are decided by its boundary and resolution; e.g., the dimensions of each floor at Finnish Geodetic Institute (FGI) are about 80 × 80 m. If a resolution of 0.01 m is selected as the accuracy of the positioning, the map cell size will be 8000 × 8000. If a larger area is investigated or if multi-resolution layers are adopted for fast searching, the resultant oversized map may be beyond the computation capability of the current onboard computer. Therefore, we propose a dynamic patch-divided likelihood map to support any large indoor environment.

**Figure 5.**
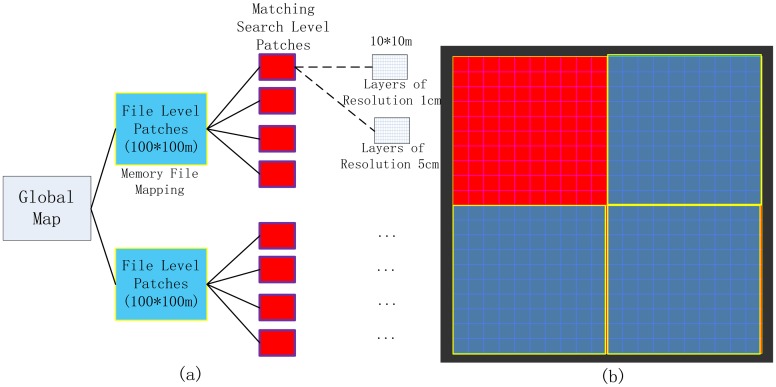
(**a**) The spatial grid-indexed likelihood map structure and (**b**) the overview map of Patch-divided data structure with colors.

The map data structure is shown in Figure 5a. The global map is first divided into file-level patches with defined areas of 100 × 100 m. According to current position and memory usage, the patches are dynamically loaded/unloaded by means of the memory-file mapping technique [34]. Then each file-level patches is divided into smaller matching-search patches shown in Figure 5a, the size of each of matching-search level patch is 10 × 10 m. The multi-resolution map layers are defined at this matching-search level. Two map layers with resolutions of 0.01 m and 0.05 m were defined for this study.

Combining matching-search patches and multi-resolution map layer techniques set restrictions on the reasonable retrieving boundary for accelerating the search speed of maximum likelihood values by reducing the amount of data retrieved. Two map layers with resolutions of 0.01 m and 0.05 m were defined for this study. All patches in the layers were indexed with the corresponding row and column ID in order to be retrieved and loaded dynamically. Figure 5b shows the related data structure in the form of an overview map with different colors.

## 3. Implementation of the System

### 3.1. System Overview

**Figure 6.**
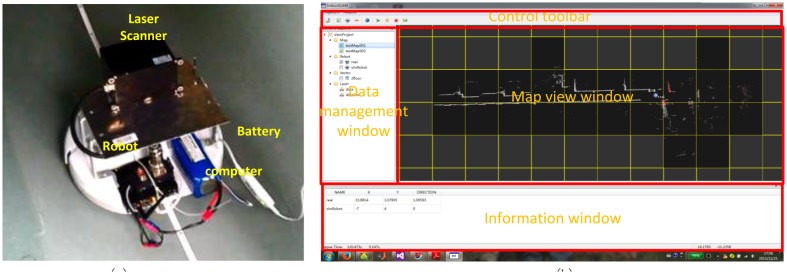
(**a**) The “NAVIS” hardware platform; (**b**) The “NAVIS” software platform.

In order to identify the simulated results and verify the proposed idea, a UGV platform called NAVIS was designed, implemented, and tested based on a home vacuum-cleaning robot (see Figure 6a)—iRobot®, which was then equipped with an odometer and determined to be capable of carrying a maximum payload of 5 kg. An “IBEO LUX” laser scanner was adopted for the tests; this device has a field-of-view of 110° with 0.25° angular resolution and a scan frequency of 25 Hz, and at maximum an effective range of 90 m indoors. The laser scanner and the robot are both connected to an onboard computer (Fit-PC-2) with its RJ-45 standard network port and COM, respectively, for collecting data, which include laser scanning data, odometer measurements, *etc.* The entire mobile unit is powered by an external battery. During the indoor tests, the robot operated at a pre-set speed of 0.28 m/s.

Software was designed and implemented for data management and positioning for NAVIS, which was implemented in the Qt SDK and Microsoft Visual Studio 2012 Integrated Development Environment (IDE) [35]. Figure 6b shows the Graphic User Interface (GUI) of the software, which is composed with four components-resource management window, map view window, information window, and the control toolbar. The resource management window manages all data resources required in the system, including likelihood maps, UGV robots, vector maps, and laser scan data; the main map view window receives the responses showing the UGV's positioning and mapping results; the numerical position and heading information is shown in the information window; and the data processing and simulation operations are manipulated using the buttons on the control toolbar.

### 3.2. Experiment Design

A thorough evaluation of our methods requires a great deal of data and experiments. Therefore, a series of simulated tests and field tests were carried out with the aforementioned multi-resolution patch-divided likelihood map.

**Figure 7.**
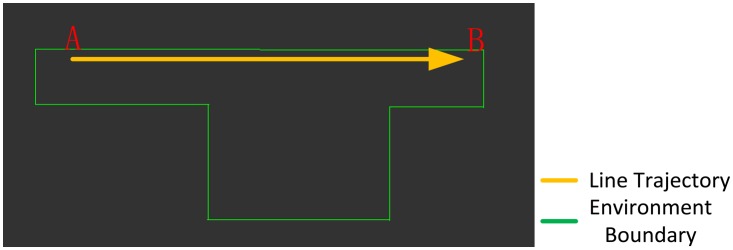
The simulated indoor environment and presetting of the UGV trajectories.

All of the simulated laser range data are created by means of a ray-cast algorithm with an angular resolution of 0.5° and a field of view of 180° based on a given vector map; we set the gauss distance noise variance at 4 centimeters when carrying out simulated range measurements; this similar to the setting for the IBEO LUX LiDAR (4 cm according to datasheet [36]) and 20 m as the maximum range with 1 cm footprint size. The structure of the simulated indoor environment is a simple T shape measuring 15 × 7 m (green line), as shown in Figure 7. A straight trajectory, represented by the orange line in Figure 7, was tested for estimating the positioning performance with the ICP, the Monte Carlo, and the IMLE algorithms in the experiment. The simulated UGV operated from location ‘A’ to ‘B’ with a steady velocity of 0.28 m/s.

**Figure 8.**
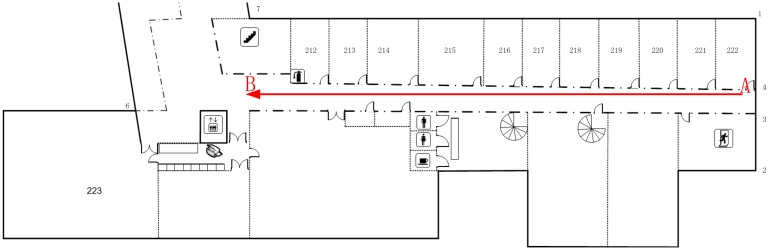
An overview map of the field test corridor and the straight trajectory.

We also discuss the positioning results of field tests based on the ICP, the Monte Carlo, and the IMLE algorithms to further verify the proposed methodology. All of the field test data were collected along a corridor on the second floor of FGI's main building. Figure 8 shows the overview map of the corridor and the experimental trajectory. The length of the experimental trajectory was approximately 38.5 m. Therefore, the area of the likelihood map could be given as 80 × 80 m considering the maximum range of the laser scanner.

The collected raw laser scan data were post-processed using the NAVIS' software on a laptop computer to evaluate the possibility of real-time application. Finally, several tests based on the field test data were also carried out to evaluate the performance of the proposed methodology with different map sizes in order to determine whether it can be used for dealing with large areas.

### 3.3. Laser Data Process

**Figure 9.**
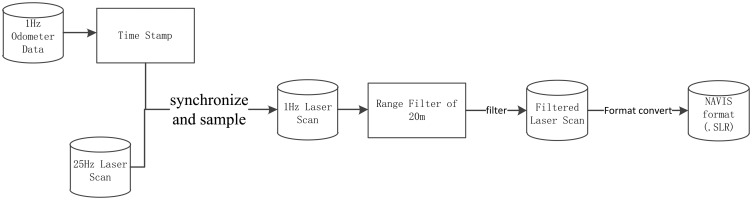
The flow chart of the laser data process.

The field test data were post-processed using Matlab and NAVIS software. Figure 9 shows the data process flow. Since the data logger records the distance traversed by the UGV at intervals of about 1 s by reading the measurement of the onboard odometer and the LiDAR sensor acquires laser scan data applying a scan frequency of 25 Hz, the laser scans are synchronized and sampled to the odometer of UGV at a time resolution of 100 ms. Following this, a filter with a range of 20 m was applied on the sampled laser data to remove the major noisy points. Finally, the filtered data were converted to the internal format of the NAVIS software for the positioning tests.

## 4. Results and Discussion

### 4.1. The Position Accuracy of ICP, Monte Carlo and IMLE with Simulated Data

**Figure 10.**
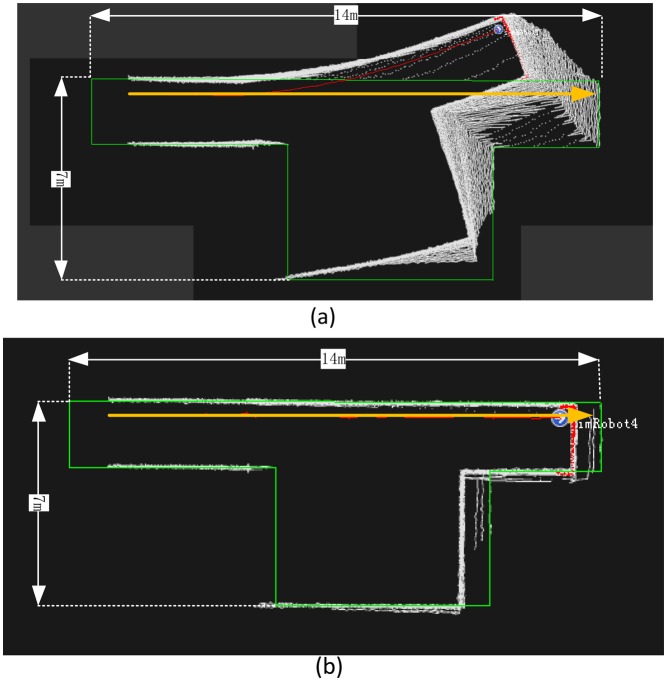

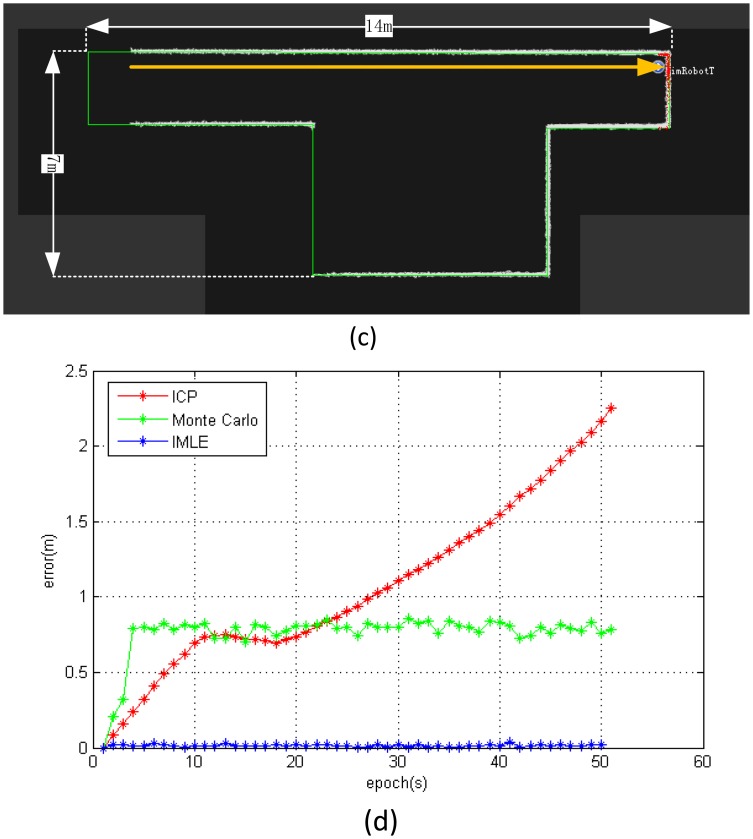
(**a**) The simulated result map with ICP at epoch 50; (**b**) The simulated result map with Monte Carlo at epoch 50; (**c**) The simulated result map with IMLE at epoch 50; and (**d**) Error comparison of ICP, Monte Carlo, and IMLE.

The position accuracy is shown in Figure 10, which demonstrates that the results of the ICP and Monte Carlo algorithm are inferior to those of the IMLE algorithm. The positioning estimation of the ICP algorithm deviates quickly when the environment changes as shown in Figure 10a. Theoretically, the Monte Carlo search is a heuristic search method. Its heading estimation maintains better accuracy when compared to ICP, but the position still deviates quickly because the inherent problem of the heuristic search method is that it always drives the result into the local optimum value as shown in Figure 10b. According to Figure 10c, the likelihood map matches the original vector map constantly, which enables the position accuracy to be at centimeter level consistently. It is then concluded that the proposed IMLE algorithm works correctly in such a feature-rich environment. The position errors of the different methods are shown in Figure 10d and the RMS errors of the ICP, Monte Carlo, and IMLE algorithms were 1.2 m, 0.77 m, and 0.02 m in local coordinate frame, respectively. Thereby, the simulated results proved that our algorithm can offer a quick and consistent positioning solution with centimeter accuracy.

### 4.2. Position Accuracy of ICP, Monte Carlo, and IMLE in Field Tests

**Figure 11.**
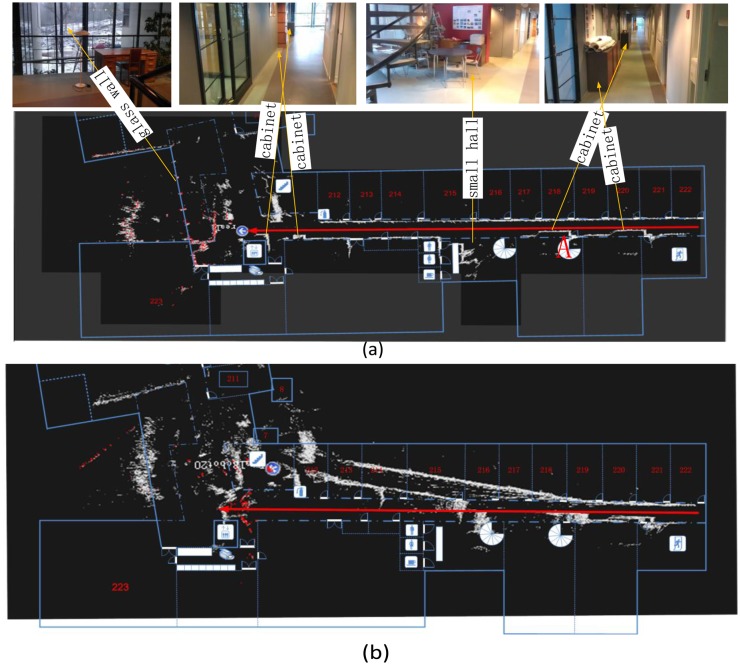

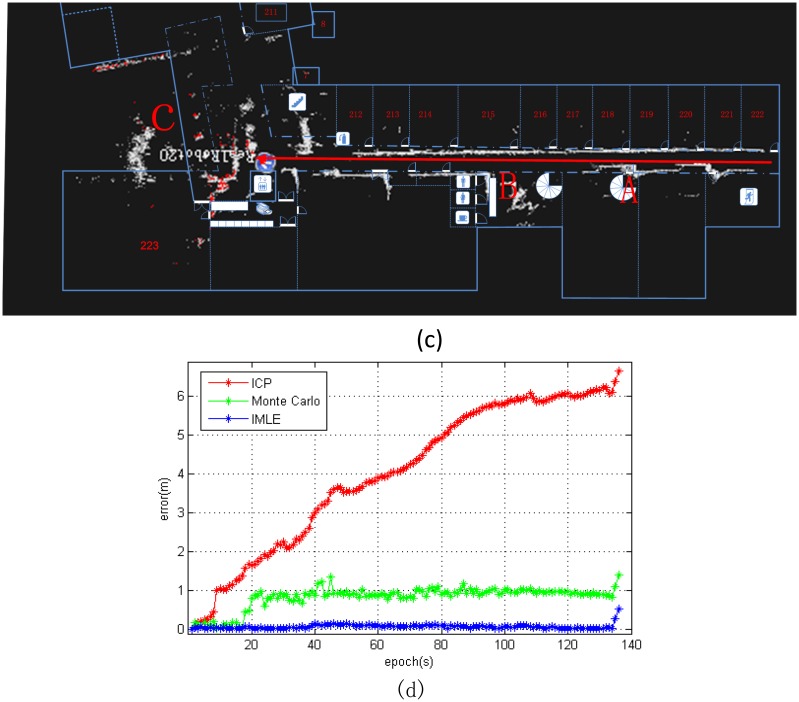
(**a**) The position and likelihood map results of the field test when using the IMLE algorithm; (**b**) The field test result map when using the ICP algorithm; (**c**) The field test result map when using the Monte Carlo algorithm; and (**d**) Error comparisons of the ICP, the Monte Carlo, and the IMLE algorithms.

Figure 11a shows the result of the likelihood map for the IMLE algorithm. Compared with the geographic map extracted from the construction blueprint, the skeleton of the corridor was accurately recognized. At the end of trajectory, the glass wall, the glass handrails, and plants caused a lot of noise measurements, which result in degraded positioning. Most of the scan points penetrated the transparent glass wall at the end of the corridor. But the line feature of the glass wall was still recognizable from the opaque window frame. Therefore, the transparency of the objects significantly affects the positioning and mapping result. Figure 11b,c shows the results of the likelihood map with the ICP and the Monte Carlo algorithms. The two algorithms behaved in the same manner as in the simulated experiment: the heading angle deviated quickly with the ICP algorithm; but the Monte Carlo algorithm easily fell into the local optimum, which caused accumulating errors and led to inadequate position results and mapping results. For example, the map feature ‘A’ in Figure 11c was much noisier than the same feature in Figure 11a, which was the local optimum position calculated by the Monte Carlo algorithm and the insufficient position resulted in incorrect mapping. Figure 11d compares the errors of the ICP, the Monte Carlo, and the IMLE algorithms in the field tests. The onboard odometer's outputs during the test were utilized as reference values. The error increased abnormally during the last few epochs on the trajectory regardless of which algorithm was adopted. The explanation for such phenomenon is the noise introduced by the glass walls and by plants, which had the effect of increasing the uncertainty of all algorithms' outputs. In those parts of the corridor with rich features for matching, the positioning RMS errors of the ICP, the Monte Carlo, and the IMLE algorithms were 4.4 m, 0.86 m, and 0.09 m. Table 1 shows the positioning error statistics. The obtained trend coincided with the simulated results. However, the accuracy of the IMLE algorithm was poorer than that of the simulation. The reason for this may be in the field of view of the selected LiDAR having been 110° against 180° in the simulation, and this means that fewer features were extracted in the field test. The difference in features between the simulation and the field test may be another reason.

In conclusion, based on the simulation results and the field test results, the UGV position system we propose in this paper can provide positioning results with decimeter accuracy at least in feature-rich indoor environments.

### 4.3. Evaluation for Real-Time Processing

**Figure 12.**
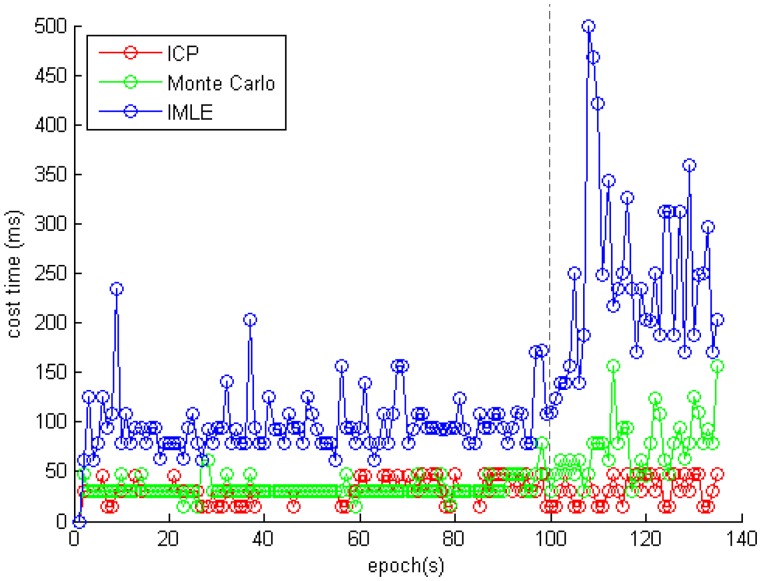
The time cost of each scan matching when using the different algorithms.

Figure 12 and Table 2 show the time cost on each scan matching when using the different algorithms. The average cost times of the ICP, the Monte Carlo, and the IMLE algorithms were 32 ms, 45 ms, and 112 ms, respectively. It is obvious that the ICP and the Monte Carlo compute about twice as fast as the IMLE on the premise of sacrificing position accuracy. And there is the phenomenon that the IMLE costs much more after about 100 s. Because there were a lot of glass features in the environment at the end of the trajectory, the introduced noisy laser scanning point cloud makes the scan matching processing more difficult. The IMLE algorithm adaptively searches for the result over a larger scope. The average cost time is about 80ms with enough matching features in 100 s. Since the UGV platform is running at a slow but steady speed of 0.28 m/s, and the positioning frequency is 1 Hz, the proposed method can finish the positioning process even under the worst conditions. The positioning frequency of the proposed NAVIS platform can reach 12 Hz in a feature-rich environment and 2 Hz even in a feature-poor environment. Therefore, it could be utilized in real-time applications.

### 4.4. Large-Area Test

**Figure 13.**
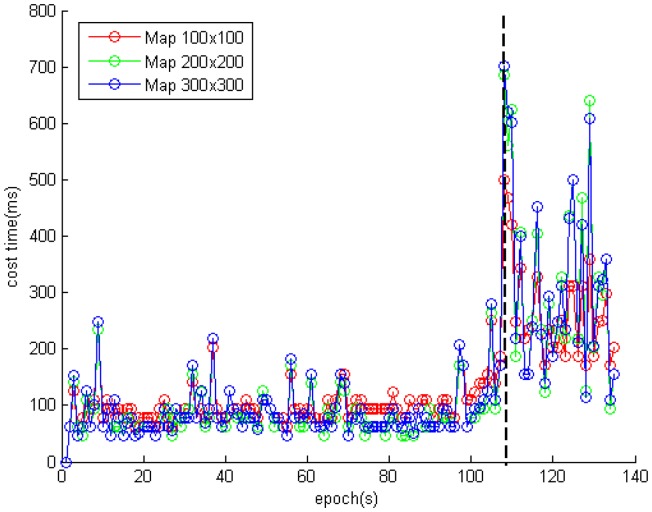
The time cost of each scan matching with different likelihood map sizes.

As is mentioned above in Section 3.5, position accuracy partly depends on the resolution of the likelihood map; the higher the accuracy, the higher the required map resolution. While the average area of a typical office building is approximately 100 × 100 m which covers most indoor mapping applications with some supermarkets being even larger, we believe that the area dealt with should be smaller 300 × 300 m. Considering 1 cm map resolution as being selected, the computer needs to be capable of storing and retrieving a huge amount of data in real-time positioning applications. The field tests conducted in the course of the present study proved that the proposed multi-resolution patch-divided likelihood map method can solve this problem. Figure 13 and Table 3 show the time cost and data amount comparisons for different likelihood map sizes with 1 cm map resolution. The data amount grows according to the Index law. However, the time cost per scan matching and map updating do not growing with increasing map size, but keeps constant at approximately 130–140 ms because the map data are loaded into memory dynamically. While there is a critical high point in Figure 13, indicated by the black dashed line, the processing time for the maps of 200 × 200 m and 300 × 300 m costs 200 ms more than that for the map of 100 × 100 m. The reason is that another map patch is need by the scan matching processing and such map loading requires extra time. Above all, the proposed map can support large-area indoor positioning and mapping in real-time applications.

## 5. Conclusions

This paper presents a real-time UGV-based positioning system called NAVIS using laser scan matching in a large indoor environment. A low-cost robot platform was selected to setup a UGV platform carrying an IBEO LUX laser scanner. The IMLE method using laser scan matching is proposed for the real-time accurate positioning of the UGV. Based on the results of simulations and field tests, it is concluded that: (1) the proposed method offers a quick and consistent positioning solution with an decimeter accuracy for large-area positioning and mapping applications; (2) in a feature-rich environment, the average latency is 80 ms, which equals 12 Hz updating frequency; and in a feature-poor environment, the corresponding value increases to 500 ms, which equals 2 Hz updating frequency. Therefore, it can be utilized in real-time applications.

The proposed IMLE algorithm heavily depends on the complexity of the environment, which may restrict its applications for different purposes. Therefore, in our future work, more sensors, such as camera, gyroscope, will be integrated to the current NAVIS to promote the system stability and thereby extend its applicability in various fields.

## Figures and Tables

**Table 1.** The position error statistics (m).

	**RMS Error**	**Mean Error**	**Maximum Error**
ICP	4.4	4.0	6.63
MONTE CARLO	0.86	0.8	1.39
IMLE	0.09	0.06	0.52

**Table 2.** Average Cost Time (ms).

	**Before Epoch 100s**	**After Epoch 100s**	**All Epochs**
ICP	31	32	32
Monte Carlo	33	79	45
IMLE	80	260	112

**Table 3.** The comparison of different likelihood map sizes with 1 cm map resolution.

**Map Size(m^2^)**	**Average Time Cost (ms)**	**Maximum Time Cost (ms)**	**Data Amount (Mb)**
100 × 100	137	499	101
200 × 200	132	687	404
300 × 300	136	702	1009
